# Supplementary material for: Regulation of atmospheric circulation controlling the tropical Pacific precipitation change in response to CO2 increases
Source: Nat Commun. 2019 Mar 7;10:1108. doi: 10.1038/s41467-019-08913-8 (PMC6405775; doi:10.1038/s41467-019-08913-8)
Supplement: Supplementary file 2 — Supplementary Information [file 41467_2019_8913_MOESM2_ESM.docx]

**Supplementary Information**

**Regulation of atmospheric circulation controlling the tropical Pacific precipitation change in response to CO_2_ increases**

^1^School of Earth and Environmental Sciences, Seoul National University, Seoul, Korea

^2^Department of Environmental Marine Science, Ansan, Hanyang University

^3^NASA, USA

*Corresponding author: Sang-Wook Yeh, Hanyang University, Ansan, Korea. E-mail: swyeh@hanyang.ac.kr*

*To be submitted to Nature Communication*

**
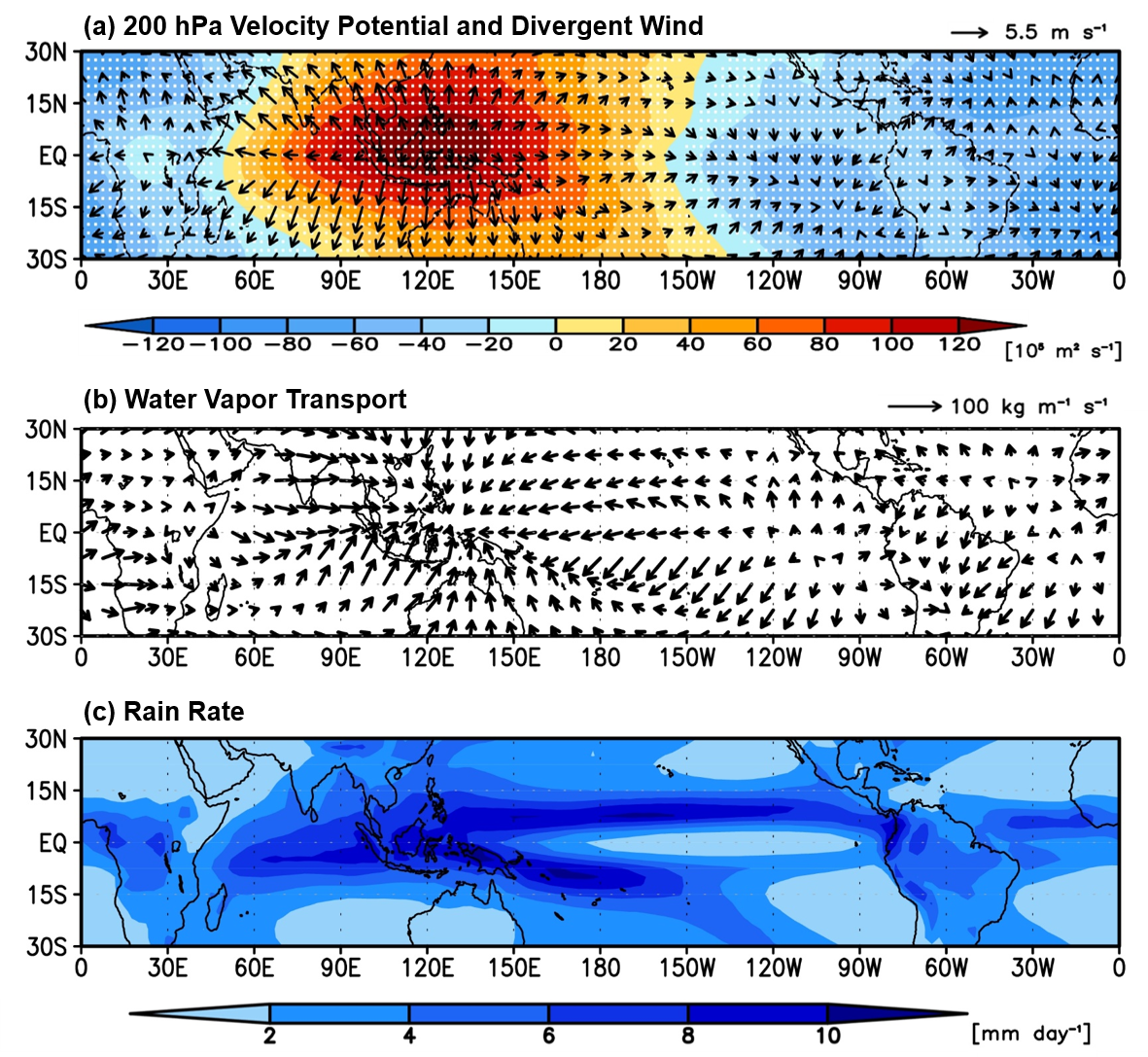
**

**Supplementary Figure 1 Mass overturning circulation and precipitation in the tropics from the 21 CMIP5 pre-industrial run** Same as in Fig. 1, but derived from the ensemble mean of 21 CMIP5 pre-industrial runs in the last 250 years simulation period.

*
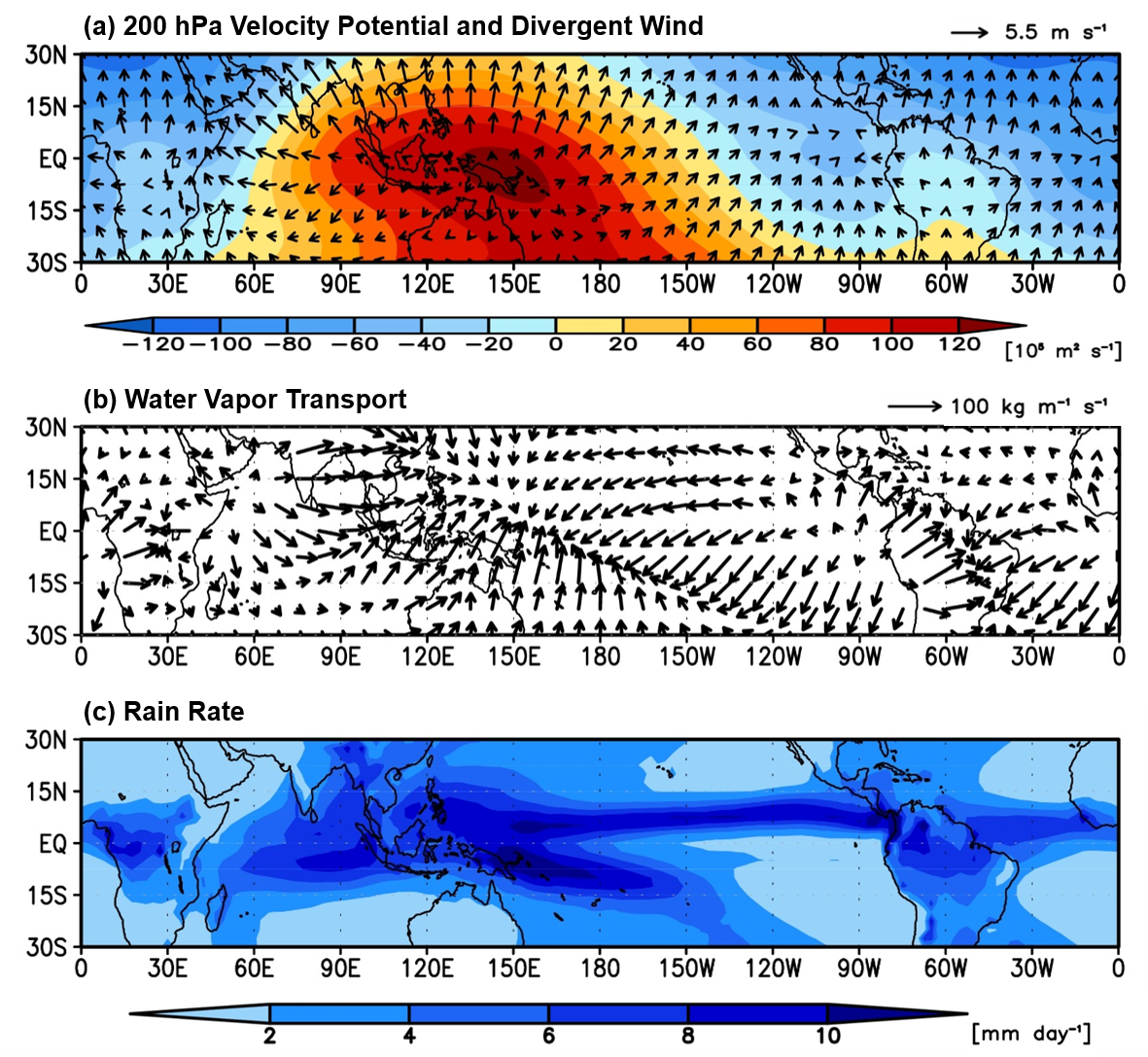
*

**Supplementary Figure 2.** **Mass overturning circulation and precipitation in the tropics in the reanalysis dataset** Same as in Figure 1, but derived from ERA-Interim reanalysis (1979-2017).


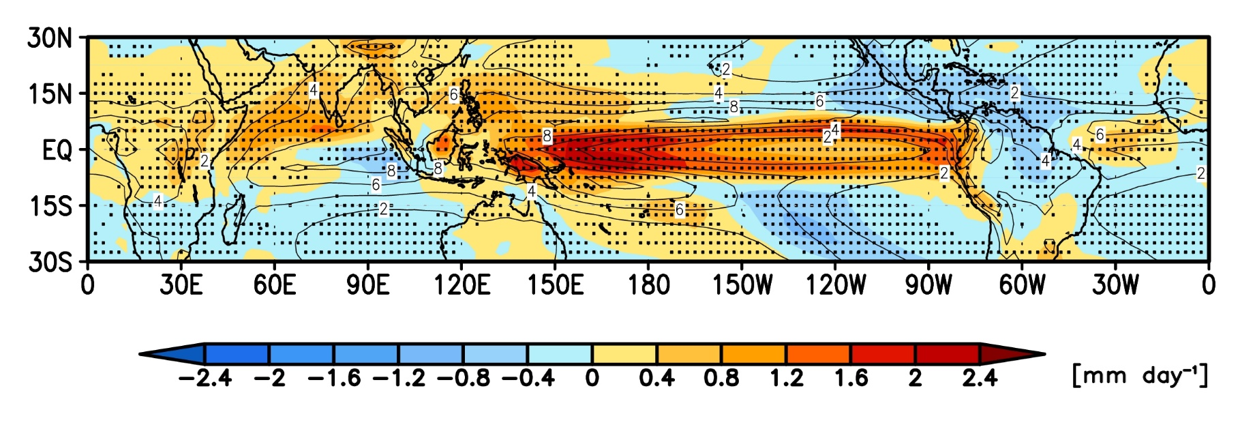


**Supplementary Fig. 3 Changes in the spatial pattern of precipitation in response to CO_2_ concentration increases** Difference of ensemble mean annual precipitation for the last 20 years (121-140 years) in the quadrupling experiment of CO_2_ concentration and the last 250 years simulation period in the CMIP5 pre-industrial run. Contours represent ensemble mean annual precipitation for the last 250 years simulation period in the CMIP5 pre-industrial run with intervals of 2 mm day^-1^. Dots denote the region where the responses of 14 or more out of the 21 CMIP5 climate models are of the same sign.


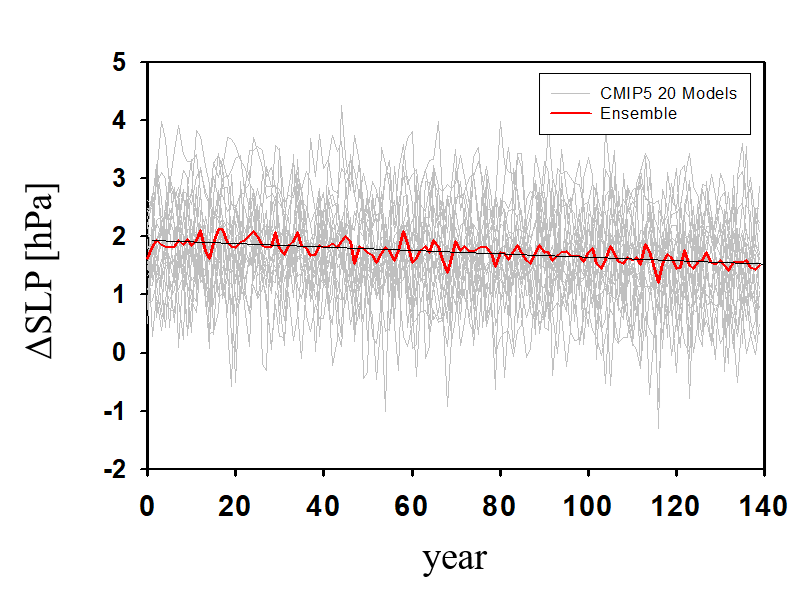


**Supplementary Figure 4. Changes in the Walker circulation intensity** Time series of Walker Circulation Index defined as sea level pressure difference between Tahiti (160°W – 80°W and 5°N – 5°S) and Darwin (80°E – 160°E and 5°N – 5°S) areas in the 21 CMIP5 CO_2_ quadrupling experiments. Unit is hPa.

**
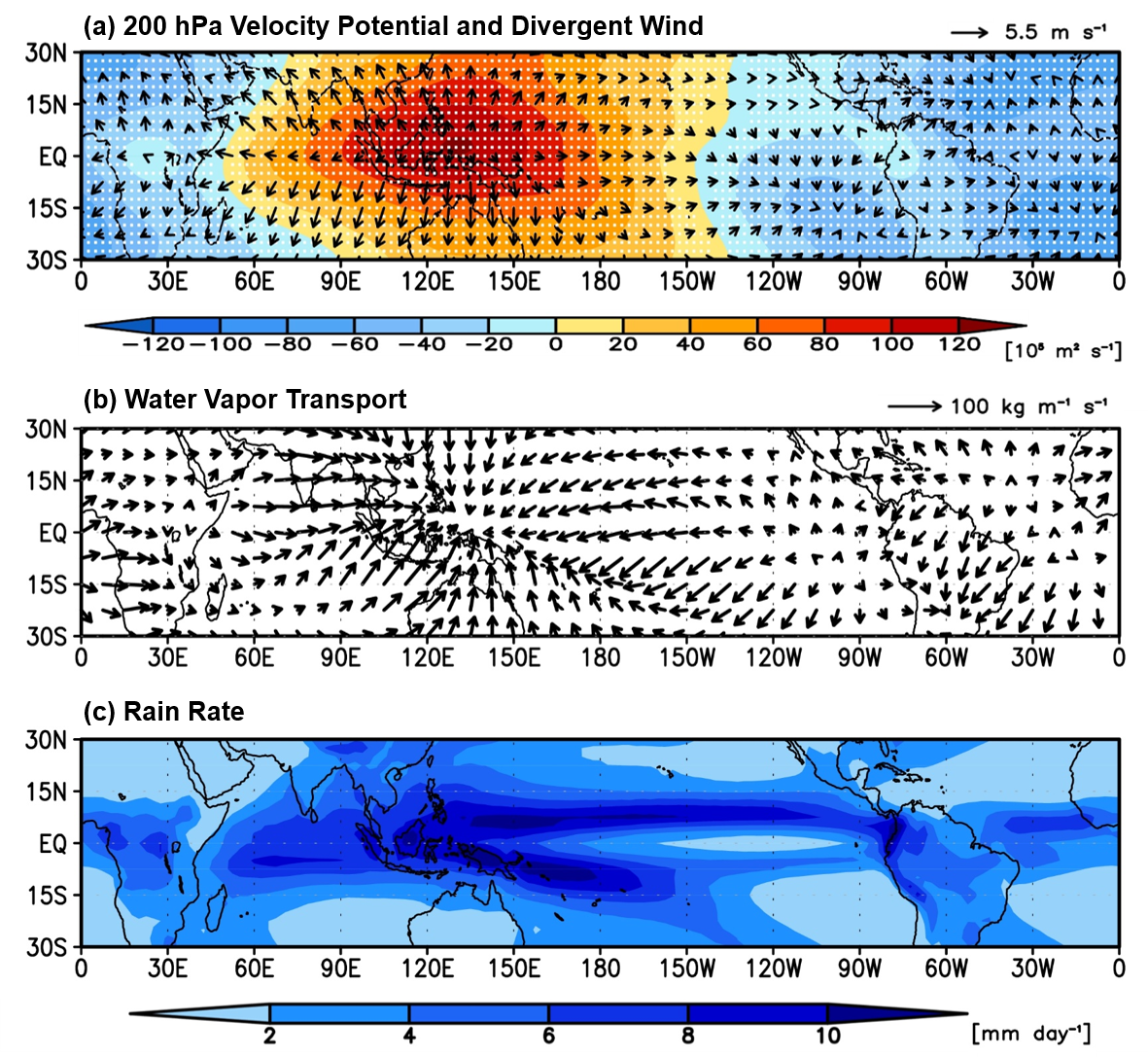
**

**Supplementary Figure 5 Mass overturning circulation and precipitation in the tropics in the quadrupling of CO_2_ concentration** Same as in Fig. 1, but for the last 20 years (121-140 years) in the quadrupling of CO_2_ concentration.


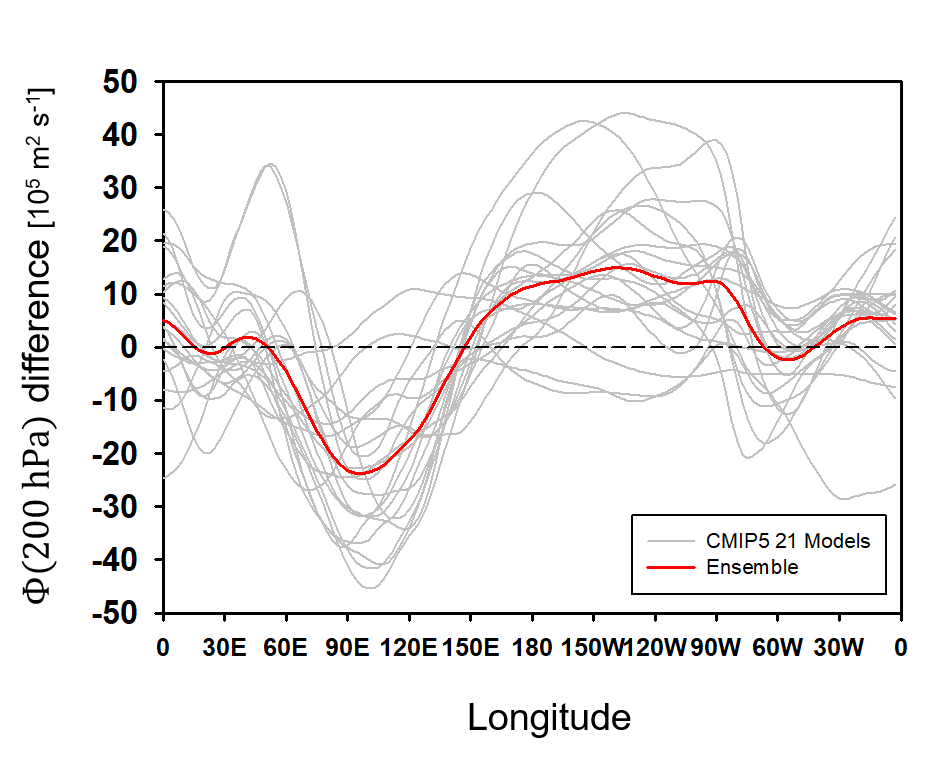


**Supplementary Figure 6** **Changes in 200 hPa velocity potential to CO_2_ concentration increases** Difference distribution (121-140 yeas minus 1-20 years) of 200 hPa velocity potential averaged in the 10°N – 10°S in each 21 CMIP5 models (gray line) and their ensemble mean (red line)**.**

**
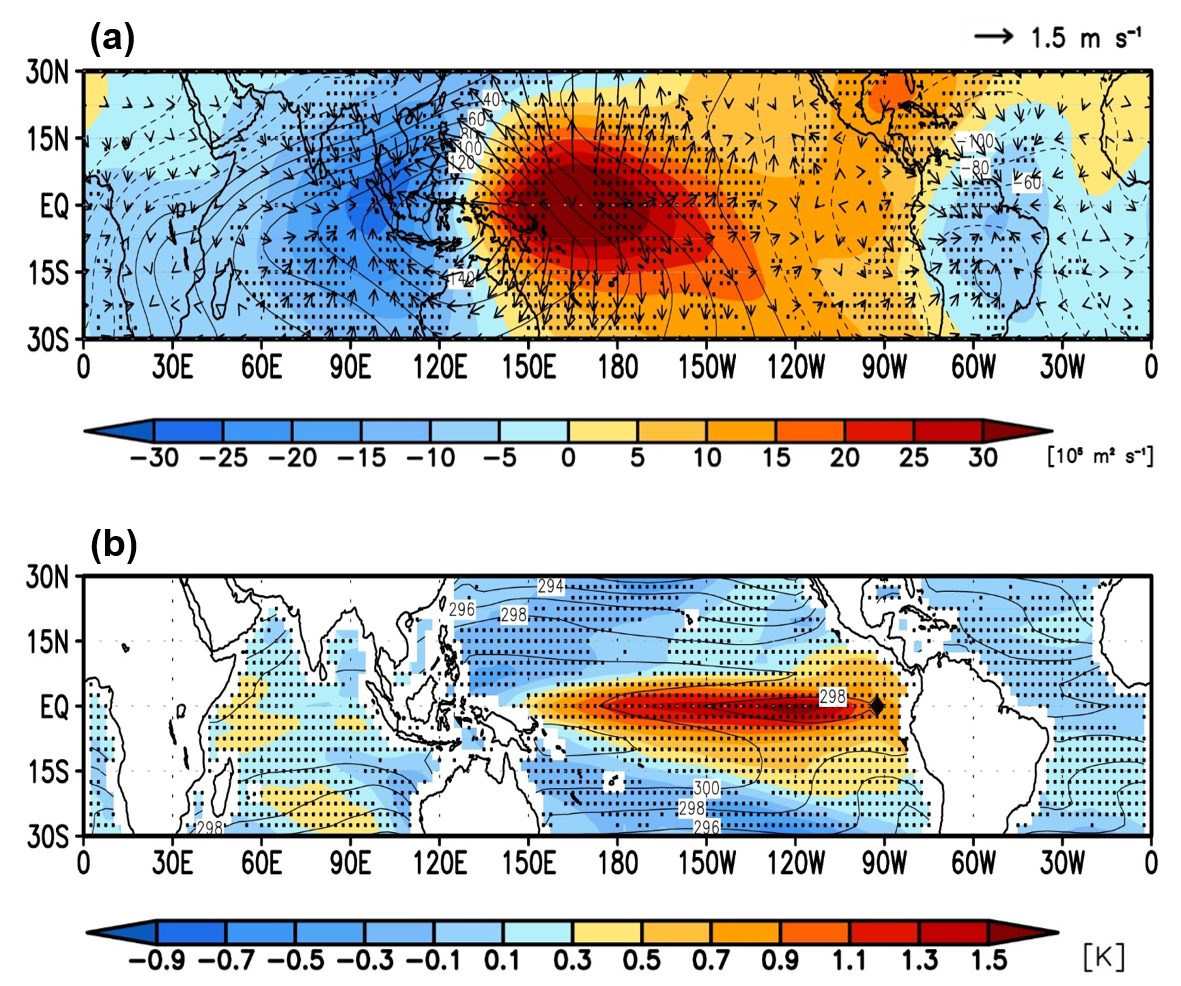
**

**Supplementary Figure 7 The composited 200 hPa velocity potential, divergent wind and SST during El Nino events** (a) The ensemble mean composited map of 200 hPa velocity potential and divergent wind in the years when El Nino occurs during boreal winter (December-January-February) in the first 20-years from 21 CMIP5 model simulation (quadrupling experiment of CO_2_ concentration). (b) is the same as in (a) except SST anomaly. Contours in (b) denotes the ensemble mean SST during boreal winter in the first 20-years from 21 CMIP5 model simulation. Dotted in (a), (b) denotes the region where the responses of 14 or more out of the 21 CMIP5 models are of the same sign.


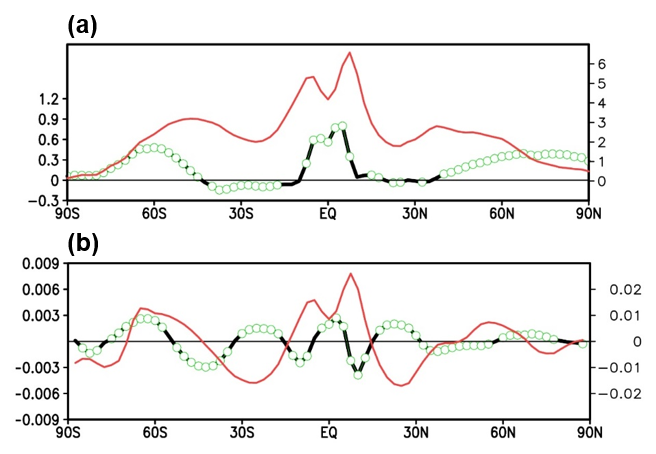


**Supplementary Figure 8 Changes in precipitation and vertical motion to CO_2_ concentration increases** Latitudinal distribution of ensemble mean of (a) annual precipitation and (b) 500 hPa vertical motion averaged in 0-360°E in the control run (i.e., 1-20 years) (red line). Black lines in (a), (b) indicate the difference of ensemble mean precipitation and 500 hPa vertical motion (121-140 years minus 1-20 years), respectively. Note that green circles denote the region where the responses of 14 or more out of the 21 CMIP5 models are of the same sign.

**
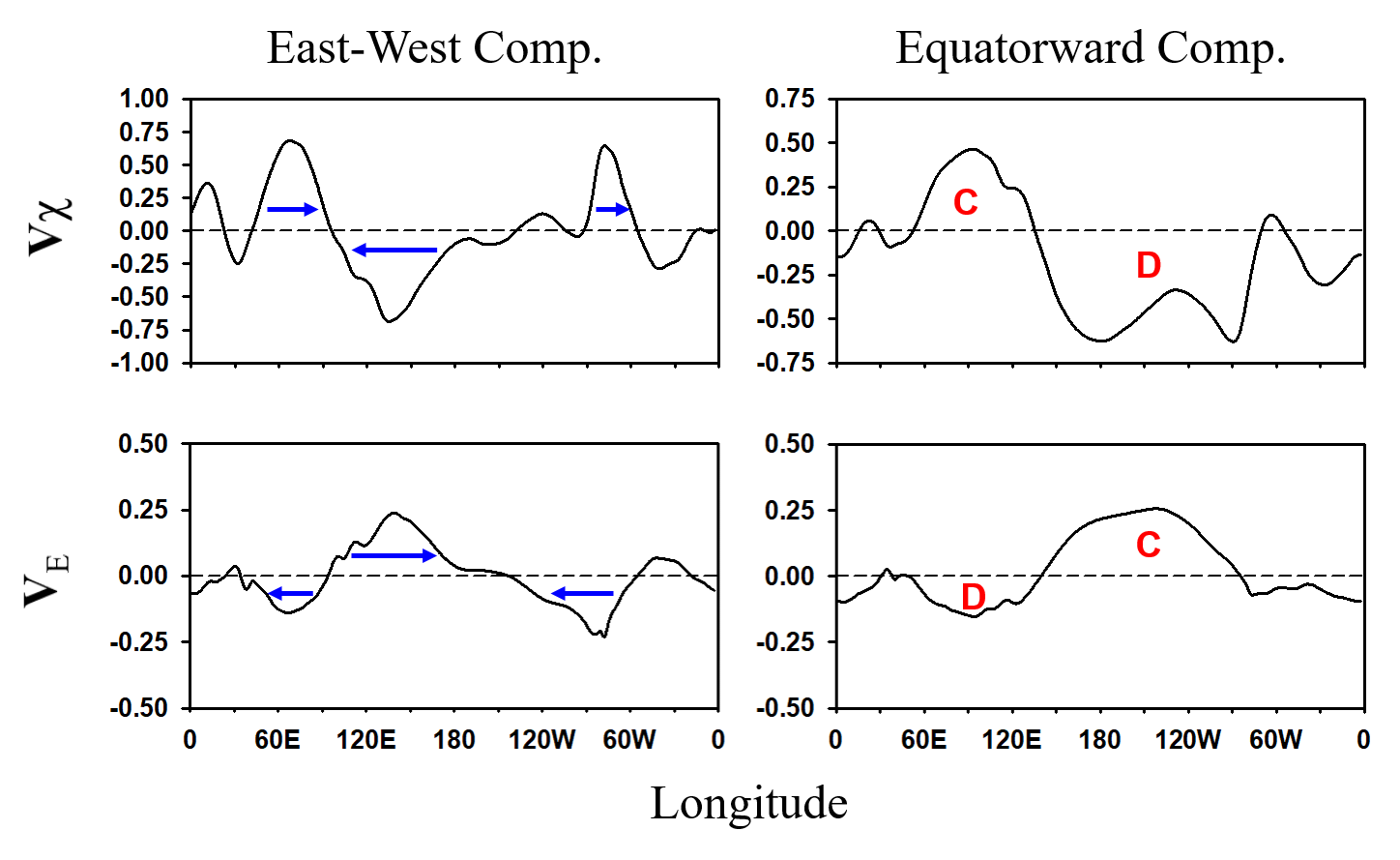
**

**Supplementary Figure 9. The tropical circulation anomalies inferred from 200 hPa divergent wind and V**_E_ Ensemble mean of (top) 200 hPa divergent wind and (bottom) effective wind [**V**_E_] over the 10°N – 10°S zonal band. Left (Right) panel shows east-west (equatorward) component of the wind. Blue arrows indicate direction of the wind and letter C (D) represents convergence (divergence).

**
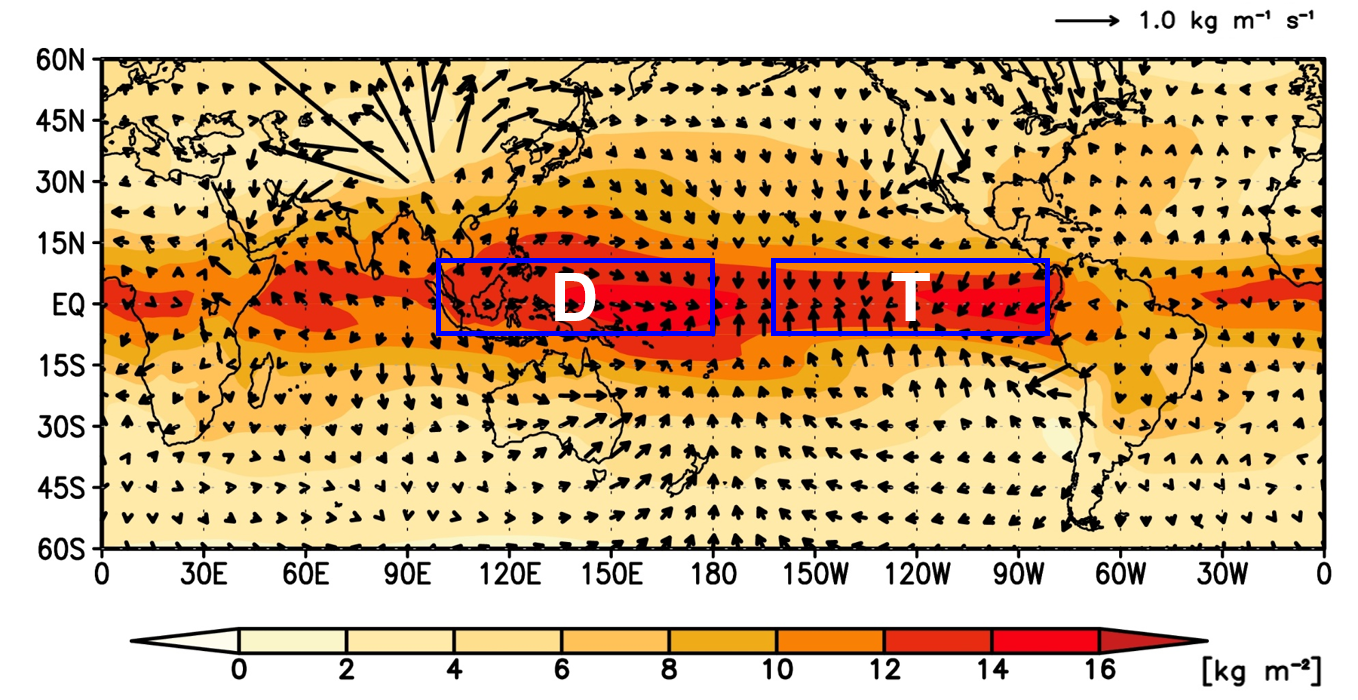
**

**Supplementary Figure 10. Changes in total precipitable water and effective wind to CO_2_ concentration increases** Global difference distribution (121-140 years minus 1-20 years) of ensemble mean of total precipitable water (colors) and effective wind (arrow). Blue boxes denote Darwin (D) and Tahiti (T) areas, respectively.

Supplementary Table 1. The 21 CMIP5 models used in this study.

Model name Horizontal resolution Vertical resolution Country / Center

(Latitude x Longitude) (No. of levels / Top pressure)

ACCESS1-0 1.25º x 1.88º 17 / 10 hPa Australia /

CSIRO&BOM

BCC-CSM1.1 2.79º x 2.81º 17 / 10 hPa China / BCC

BNU-ESM 2.79º x 2.81º 17 / 10 hPa China / GCESS

CNRM-CM5 1.40º x 1.40º 17 / 10 hPa France /

CNRM-CERFACS

CSIRO-Mk3.6.0 1.86º x 1.88º 18 / 5 hPa Australia /

CSIRO

CanESM2 2.79º x 2.81º 22 / 1 hPa Canada / CCCma

GFDL-CM3 2.02º x 2.50º 23 / 1 hPa USA / NOAA

GFDL-ESM2G 2.02º x 2.50º 17 / 10 hPa USA / NOAA

GFDL-ESM2M 2.02º x 2.50º 17 / 10 hPa USA / NOAA

HadGEM2-ES 1.25º x 1.88º 17 / 10 hPa UK / MOHC

INMCM4 1.50º x 2.00º 17 / 10 hPa Russia / INM

IPSL-CM5A-LR 1.89º x 3.75º 17 / 10 hPa France / IPSL

IPSL-CM5A-MR 1.27º x 2.50º 17 / 10 hPa France / IPSL

MIROC-ESM 2.79º x 2.81º 35 / 0.03 hPa Japan / MIROC

MIROC5 1.40º x 1.40º 17 / 10 hPa Japan / MIROC

MPI-ESM-LR 1.86º x 1.88º 25 / 0.1 hPa Germany /

MPI-M

MPI-ESM-P 1.86º x 1.88º 25 / 0.1 hPa Germany /

MPI-M

MRI-CGCM3 1.11º x 1.13º 23 / 0.4 hPa Japan / MRI

NCAR CCSM4 0.94º x 1.25º 17 / 10 hPa USA / NCAR

NorESM1-M 1.89º x 2.50º 17 / 10 hPa Norwegian /

NCC

NorESM1-ME 1.89º x 2.50º 17 / 10 hPa Norwegian /

NCC
